# Regression applied to legal judgments to predict compensation for immaterial damage

**DOI:** 10.7717/peerj-cs.1225

**Published:** 2023-03-23

**Authors:** Thiago Raulino Dal Pont, Isabela Cristina Sabo, Jomi Fred Hübner, Aires José Rover

**Affiliations:** 1 Department of Automation and Systems, Universidade Federal de Santa Catarina, Florianopolis, Santa Catarina, Brazil; 2 Department of Law, Universidade Federal de Santa Catarina, Florianopolis, Santa Catarina, Brazil

**Keywords:** Text regression, Immaterial damage compensation, Natural language processing, Consumer law, Brazilian legal judgments

## Abstract

Immaterial damage compensation is a controversial matter in the judicial practice of several law systems. Due to a lack of criteria for its assessment, the judge is free to establish the value based on his/her conviction. Our research motivation is that knowing the estimated amount of immaterial damage compensation at the initial stage of a lawsuit can encourage an agreement between the parties. We thus investigate text regression techniques to predict the compensation value from legal judgments in which consumers had problems with airlines and claim for immaterial damage. We start from a simple pipeline and create others by adding some natural language processing (NLP) and machine learning (ML) techniques, which we call adjustments. The adjustments include *N-Grams Extraction, Feature Selection, Overfitting Avoidance, Cross-Validation* and *Outliers Removal*. An special adjustment, *Addition of Attributes Extracted by the Legal Expert (AELE)*, is proposed as a complementary input to the case text. We evaluate the impact of adding these adjustments in the pipeline in terms of prediction quality and execution time. *N-Grams Extraction* and *Addition of AELE* have the biggest impact on the prediction quality. In terms of execution time, Feature Selection and Overfitting Avoidance have significant importance. Moreover, we notice the existence of pipelines with subsets of adjustments that achieved better prediction quality than a pipeline with them all. The result is promising since the prediction error of the best pipeline is acceptable in the legal environment. Consequently, the predictions will likely be helpful in a legal environment.

## Introduction

Immaterial damage compensation is one of the most controversial matters in the judicial practice of several law systems due to a lack of criteria for its assessment. Consequently, the judge is free to evaluate and define the amount of compensation based on his/her internal conviction (Sadiku, 2020).

In the Brazilian justics system, for example, the compensation for immaterial damage in Consumer Law problems is a subject often discussed in the Special Courts. Also, statistics indicate an increase of 6.8 percent in new cases in 2019, the highest number recorded until then in the local practice (CNJ, 2020). This rise of immaterial damages lawsuits stems from the empowerment of the population in terms of citizenship, who become aware of their rights and exercise them (Melo, 2012; Boselina, 2019).

However, it impacts negatively on the time that a lawsuit takes to be judged. In view of this high litigation and the lack of criteria about immaterial damage, Machine learning (ML) is a potential technique to modernize the courts around the world and assist in the consistency of decisions (Peixoto & Silva, 2019), including the compensation values.

In this context, our research question is: To what extent the prediction of compensation values can be *accurate* and *helpful* in the legal environment using regression models? We answer this question by evaluating the gains of applying natural language processing (NLP) and machine learning techniques on regression pipelines used to predict the values, called adjustments. The adjustments include *N-Grams Extraction* (pre-processing step), *Feature Selection* (representation step), *Overfitting Avoidance* (regression step), *Cross-Validation* and *Outliers Removal* (training step). A special adjustment, *Addition of Attributes Extracted by the Legal Expert (AELE)*, is proposed as a complementary input, whose gains we also evaluate.

The results are based on experiments from a dataset of 928 legal judgments in which consumers had problems with airlines and received compensation for immaterial damage. The first part of the question (accuracy) is assessed by regression prediction quality metrics and the second part (helpfulness) by the legal expert’s experience.

The research motivation is that by informing the parties the estimated amount of immaterial damage compensation at the initial stage of a lawsuit can encourage an agreement between them.

## Related work

According to our literature review, using Scopus, IEEE Xplore and ACM, there is only one published research on the application of regression on legal texts. Thus, this section also considers publications for other areas.

Regarding the legal domain, Yeung (2019) trained a bidirectional encoder representations from transformers (BERT) model based on a German legal *corpus*. The author applied such representation to downstream tasks, such as classification and regression. In the former, the author tried to classify the cases according to their jurisdiction and level of appeal. In the latter, the author implemented a model to predict compensation values based on textual documents and linear regression. The author compares the BERT model with term frequency-inverse document frequency (TF-IDF) and with FastText. The results for the classification task show that using BERT trained in German legal texts yields results better than TF-IDF, but comparable to FastText and BERT from general German texts. However, in the regression task, TF-IDF yields the best results when compared to the FastText and BERT models. Thus, results show that more complex representations were not suited for the regression task in the author’s dataset.

Joshi et al. (2010) used text regression to predict a movie’s opening weekend revenue. They collected data for movies released in 2005–2009. For these movies, they obtained metadata and a list of hyperlinks to movie reviews by MetaCritic, and each movie’s production budget, opening weekend gross revenue, and the number of screens on which it played during its opening weekend from The Numbers. They applied linear regression combined with N-Grams. The results revealed that review text can replace metadata and even improve the prediction quality.

Lampos et al. (2014) used text regression to predict a user impact score, estimated by combining the numbers of the user’s followers, followees and listings. They formed a Twitter dataset of more than forty-eight million tweets produced by 38,020 users located in UK in the period between April 14, 2011, and April 12, 2012. They applied linear as well as nonlinear learning methods (Gaussian process). The results generated strong predictions, especially with models based on the Gaussian process, and showed that activity, non-clique-oriented interactivity, and engagement on a diverse set of topics are among the most decisive impact factor.

Trusov et al. (2015) used text regression to predict next year change in stock price volatility in the context of financial risk problems. They collected data from traded companies’ reports provided by the EDGAR system, maintained by the U.S. Securities and Exchange Commission (SEC), and stock prices *via* Yahoo Finance. They applied Support Vector Regression and Random Forest models associated with Bag of Words representation with Latent Dirichlet Allocation (LDA) and TF-IDF. The results showed that models with multiple representations outperform single representation models.

Zou et al. (2016) used text regression to detect and quantify infectious intestinal diseases (IIDs) from social media content. They collected Twitter data and social health surveillance records obtained from Public Health England (PHE) and applied a regularized linear (elastic net) as well as a nonlinear (Gaussian process) regression function for inference. The results indicated that both in terms of prediction quality and semantic interpretation, Twitter data contain a signal that could be strong enough to complement conventional methods for IID surveillance. In regard to text regression, the nonlinear approach performs better.

Kusmierczyk & Nørvåg (2016) used text regression to predict nutritional fact values of an unknown recipe within the context of dietary pattern analysis in food-focused social networks. They collected data from the largest English online food recipe platform, namely *allrecipes.com*. Each recipe has a title and information about nutritional facts (per 100 g). They applied LDA with linear regression and with gradient boosted regression trees. The experiments showed the extent to which it is possible to predict nutrient facts from meal names.

Xu & Lee (2020) used text regression to analyze online consumer reviews and managerial responses from the hotel industry. They collected online consumer reviews about the well-known Marriot Hotel chain from three platforms, namely Expedia, representing third-party booking platforms; TripAdvisor, representing social-media platforms; and the Marriot’s official booking platform, representing direct platforms (channels). They applied multinomial logistic regression combined with latent semantic analysis (LSA) and TF-IDF. The results suggested that although consumers have different linguistic styles and focus on different attributes in their reviews on the three platforms, the antecedents of their overall satisfaction are the same: room, employees and services, location and access, and operations and facilities. Moreover, managers differentiate between consumers’ perceptions in their review process and their perceptions about the consumption experience. Based on these results, they made recommendations for managers to provide suitable responses to the different platforms online and to improve consumer overall satisfaction.

Finally, we could find only one research directly related to ours in terms of text regression applied to the legal domain. While the work applies one regression technique, ours explores a greater variety of NLP and ML techniques in text regression pipelines.

## Dataset context and construction

The dataset is composed of 928 legal judgments issued between February 2011 to September 2020 into the State Special Court located at the Federal University of Santa Catarina. For context, the Special Courts are agencies of the Brazilian Judiciary whose purpose is to improve the citizen’s access to justice. Unlike ordinary courts, these provide facilities such as remission of lawsuit costs, procedural simplification, and incentive to conciliation between the parties. The Special Courts address daily and little conflicts (Watanabe, 1985).

The legal judgments refer only to cases in which consumers had problems with airlines. To solve them, the Code of Consumer Protection provides basic consumer’s rights, such as effective compensation for material and immaterial damages, and ways to facilitate consumer’s defense and ensure their rights. One of these is through Special Courts since it offers an unbureaucratic way out to solve their problems (Brazil, 1990).

Immaterial damage is an injury to personality rights, such as honor, dignity, intimacy, image, and name (Gonçalves, 2020). In regard to the failures in air transport service, for example, flight delay, flight cancellation or baggage loss, the courts have been decided that they can generate immaterial damage and consumer compensation (Benjamim, 2015).

Compensation for immaterial damage is usually monetary. It is not possible to evaluate the painful sensation experienced by the injured person. As a means of mitigating the consequences, money can play a satisfactory role (Diniz, 2020). There are some circumstances considered by the judge when fixing the value, such as the person’s age, health status, person’s gender, place, and time of injury. Anyway, these variables are weighted by the judge in a free assessment, according to his/her interpretation of each case (Sadiku, 2020).

To construct the dataset, a legal expert collected all the documents to avoid repeated judgments or judgments about a subject not related to failures in air transport service. The legal expert also manually extracted some attributes and their values from each document, which was possible through a clustering step (Sabo et al., 2021). One of the attributes identified, for example, is the flight delay period. Therefore, the expert analyzed every judgment and extracted the value of this attribute (the delay hours).

A legal judgment is an unstructured textual document and refers to the final decision of a lawsuit in first degree. Generally, it consists of three elements (Brazil, 2015): (1) *Report* (summary of what happened according to the parties allegations and evidences); (2) *Reasoning* (reasons that formed the judge’s conviction); and (3) *Result* (value fixed by the judge for immaterial damage compensation).

To evaluate the model, we remove the part of the document that refers to the result of the judgment since it contains the value of compensation for immaterial damage. That way, the models predicts the compensation value based on the report and the reasoning. The result is however used in the training phase as label for the example. The dataset contains a vocabulary of 16,924 words, 712,057 total tokens and an average of 758 tokens per document (after the preprocessing step). The labels (compensation values) vary from 304 to 25,000 Brazilian Reais with an average of 6,344 and a standard deviation of 3,471.

To preserve the parties privacy, the dataset was also anonymized by removing parties names and the lawsuit’s ID using regular expressions. Even though the judgments are public, the access to them in the electronic process systems is protected with *captcha* and similar tools. Considering this, we anonymized them as a good practice concerning the Brazilian data protection law, intending to avoid any further inappropriate processing of this personal data.

It follows the list of the attributes together with an explanation of their importance for the prediction problem.

- **Date of judgment:** The judge’s perspectives may change over time. Consequently, the amount of compensation may vary by date. In the dataset, this is represented by day, month, and year.
- **Judge:** Each judge is free to set the amount of compensation according to his/her conviction on the case. In this sample period, the judgments were elaborated by different judges. In the dataset, this is represented by the name of the thirty one judges who prepared the collected judgments.
- **Type of judge:** In the State Special Courts, there are three types of judges: chief, assistant, and voluntary. The chief judge is responsible for the court and is the one who, as a rule, judges the lawsuits. The assistant or substitute judge is the one who judges when the chief judge needs to be absent. And the voluntary judge is the one who has a law degree but is not invested in the position. He or she voluntarily prepares judgments that are submitted to the approval of the chief judge. An assistant judge can freely fix a different value of compensation than a chief judge. The voluntary judge can do this too, but the chief judge can modify the value. In the dataset, this is represented by the type of the judge, of which there are three.
- **Permanent baggage loss:** It is an event that can generate compensation for immaterial damage. In the dataset, this is represented by “yes” (when there was a loss) and “no” (when there was no loss).
- **Tampered baggage:** Depending on the level of damage or in case of missing consumer’s belongings (theft), it is an event that can generate compensation for immaterial damage. In the dataset, this is represented by “yes” (when there was tampering) and “no” (when there was no tampering).
- **Temporary baggage loss:** It is an event that can generate compensation for immaterial damage. In the dataset, this is represented by “yes” (when there was a loss) and “no” (when there was no loss).

– **Loss interval: **It is a sub-attribute. The longer the delay in returning the baggage to the consumer, the greater can be the value of the compensation for immaterial damage. In the dataset, this is represented by days.

- **Flight cancellation:** It is an event that can generate compensation for immaterial damage. We consider as flight cancellation those cases with no rebooking or when the destination is changed. In the dataset, this is represented by “yes” (when there was cancellation) and “no” (when there was no cancellation).
- **Flight delay:** It is an event that can generate compensation for immaterial damage. We consider as flight delay those cases with rebooking. In the dataset, this is represented by “yes” (when there was a delay) and “no” (when there was no delay).

– **Delay interval: **It is a sub-attribute. The longer the delay in rebooking (that is, the longer the interval between the initially contracted flight and the actual flight operated), the greater can be the value of the compensation for immaterial damage. In the dataset, this is represented by hours and minutes.

- **Adverse weather conditions:** It is an event that excludes the possibility of compensation for immaterial damage because it is an unpredictable situation. Even the airline’s effort is not capable of overcoming them, so there is no way to impute liability to it. In the dataset, this is represented by “yes” (when there was proven bad weather) and “no” (when there was no proven bad weather).
- **Consumer fault:** It is an event that excludes the possibility of compensation for immaterial damage because it removes the airline’s liability. An example of this situation is when the consumer does not arrive at the airport in plenty of time to check his/her flight and bags. In the dataset, this is represented by “yes” (when there was a consumer fault) and “no” (when there was no consumer fault).
- **Overbooking:** Selling more tickets for a flight than are available is considered an abusive practice. Thus, it is an event that can generate compensation for immaterial damage. In the dataset, this is represented by “yes” (when there was overbooking) and “no” (when there was no overbooking).
- **No show:** Cancellation of the return ticket unilaterally when the consumer does not show up on the outward flight is considered an abusive practice. Thus, it is an event that can generate compensation for immaterial damage. In the dataset, this is represented by “yes” (when there was cancellation by no show) and “no” (when there was no cancellation by no show).
- **Right to regret and repayment claim:** Hindering the consumer’s repayment when he/she decides to cancel the acquired ticket is an event that can generate compensation for immaterial damage. This situation is known by a sequence of bad experiences (called **via* crucis* by judges) that the consumer must face to get the repayment. In the dataset, this is represented by “yes” (when repayment was hindered) and “no” (when the repayment was not hindered or when there was no claim).
- **Downgrade:** The airline changes a business class passenger to economy class. Besides a breach of contract, it is also a breach of the consumer’s expectation, and, therefore, it is an event that can generate compensation for immaterial damage. In the dataset, this is represented by “yes” (when there was a downgrade) and “no” (when there was no downgrade).

## Regression applied to text data

The regression task is a supervised learning approach that, based on samples of pairs $(x,y)$, aims to find a function $f$ that predicts a continuous dependent variable $y$ from $x$ ( $y = f(x)$). Since $f(x)$ may not achieve a perfect mapping from $x$ to $y$, there will be some amount of error, which we want to keep as small as possible (Draper & Smith, 1998). The representations of $x$ can vary in each context. When applying regression on textual data, $x$ can be books, legal documents, *etc*., (Aggarwal, 2018).

**Figure 1:**
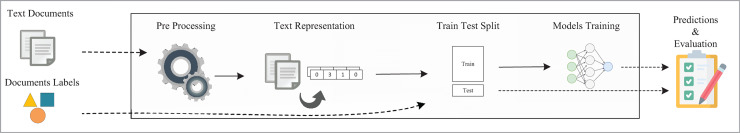
Simple regression pipeline.

As shown in Fig. 1, one can use texts in a regression task to predict one or more dependent variables (Ngo-Ye & Sinha, 2014). However, due to the unstructured nature of textual data, some specific steps to apply machine learning in texts have been included, as we discuss in the following paragraphs (Aggarwal, 2018).

The pipeline from Fig. 1 receives as inputs the textual documents and their labels. To prepare this data to further use, some preprocessing operations were applied (García, Luengo & Herrera, 2015), which include tokenization, normalization, filtering, and others (Lee et al., 1999; Jurafsky & Martin, 2019; Kotu & Deshpande, 2019).

The next step is to transform the text into a numerical representation which will serve as inputs to regression models. Among the available techniques, there is the Bag of Words (BOW) model, which transforms each document to a sequence of numbers (Kowsari et al., 2019). The numbers represent some information about each word in the text, for example, the term frequency (TF) (Baeza-Yates & Ribeiro-Neto, 1999). Beyond BOW model, there are word embeddings (Pennington, Socher & Manning, 2014; Bojanowski et al., 2016), topic modeling (Blei, Ng & Jordan, 2003; Kherwa & Bansal, 2017), and many others (Devlin et al., 2018; Peters et al., 2018; Brown et al., 2020; Pittaras et al., 2020; Dhanani, Mehta & Rana, 2022; Martino, Pio & Ceci, 2021; Chalkidis et al., 2020).

With the numerical representations of the text and their labels, the data can be split into two new datasets: train and test, comprising, for instance, eighty percent and twenty percent of the data, respectively. Models are trained using the train set and the regression techniques. Using these models, one can make predictions on some continuous output (Kotu & Deshpande, 2019).

Among regression techniques available, there are linear-based techniques such as Linear Regression (Hastie, 2009) and its derivatives, Ridge (Hoerl & Kennard, 1970), elastic net (Zou & Hastie, 2005) and Lasso (Tibshirani, 1996). Techniques based on decision trees (Breiman et al., 2017) can be used, such as Random Forest (Breiman, 2001), Gradient Boosting (Friedman, 2000), Bagging (Breiman, 1996), Adaboost (Schapire, 1999), and XGBoosting (Chen & Guestrin, 2016). Beyond linear and tree-based models, support vector machines (SVM) (Drucker et al., 1997) and neural networks (Kingma & Ba, 2015) can be adapted for the regression task.

Due to the inner differences among the regression techniques, they can achieve better or worse performances in different situations. Thus, it may be useful to apply some of those models together, so they complement one another. The final prediction of this combination is the average output among the models. This approach is called Ensemble Voting (Mendes-Moreira et al., 2012).

Considering again Fig. 1, at the final step, the estimation of the prediction quality of the models on the test set is carried out using metrics for the regression. A common metric is Root Mean Square Error (RMSE), which represents the average error of the square differences between the predicted ( ${y_i}$) and the actual ( ${\hat y_i}$) values (Aggarwal, 2018), as shown in Eq. (1). This metric is more sensitive to outliers and tends to penalize more the bigger errors (Chai & Draxler, 2014).

(1) $$RMSE = \sqrt {{{\sum\nolimits_{i = 1}^n {{{({y_i} - \hat {{y_i}})}^2}} } \over n}}$$

Another metric is the Mean Absolute Error (MAE), which represents the average of the errors when predicting the dependent variable. MAE is also simple to interpret and it is less sensible to outliers than RMSE (Chai & Draxler, 2014).

(2) $$MAE = {{\sum\nolimits_{i = 1}^n | {y_i} - \hat {{y_i}}|} \over n}$$

An additional metric is the coefficient of determination, or ${\rm R^2}$, interpreted, as shown in Eq. (3), as the proportion of observed variation in $y$ that can be explained by the regression model. So, the higher ${\rm R^2}$, the better the model can explain the variation in $y$ (Devore, 2011).

(3) $${\rm R^2} = 1 - {{\sum\nolimits_{i = 1}^n {{{({y_i} - {{\hat y}_i})}^2}} } \over {\sum\nolimits_{i = 1}^n {{{({y_i} - \bar y)}^2}} }}$$

When dealing with supervised learning applications, one can face some difficulties to get good results (Alzubaidi et al., 2021; Kornilova & Bernardi, 2021). In text mining applications, it is not different. When using the BOW model, some semantic and syntactic information is lost when considering words individually (Mikolov et al., 2013). The extraction of N-Grams from the text can thus contribute to reducing this problem. N-Grams are sequences of *N* words that appear consistently in the text. In the BOW representation, each extracted N-Gram is a single unit (Aggarwal & Zhai, 2012).

Another challenge is the *overfitting*. It occurs when the models are too specialized in the train data and they achieve a poor prediction quality when evaluated in the test set (Karystinos & Pados, 2000). According to Hawkins (2004), a model is overfitted when it achieves the same prediction quality when compared to a simpler one. Thus, the model is more complex than it should be. A possible adjustment to reduce overfitting is to reduce the complexity of the models, that is, check whether simpler models perform as well as the complex ones (Liu & Gillies, 2016).

The input dimensionality also has an impact on the overfitting problem (Liu & Gillies, 2016). Considering textual data representation from Bag of Words, for instance, the input can have dozens of thousands of words, and many words and N-Grams appear a few times in the text (Aggarwal, 2018). In this context, the use of feature selection techniques can improve the text representation and the prediction quality (Chandrashekar & Sahin, 2014).

A further common challenge that can affect the learning task is the presence of outliers in the dataset. Although the feature selection techniques make improvements in the input representation (Miao & Niu, 2016), there may be instances very distinct or inconsistent from the others. These instances are called *outliers*. Their existence in the dataset may degrade the prediction quality of the models (Freeman, Barnett & Lewis, 1995). Among the existing algorithms for discovering outliers (Hodge & Austin, 2004), there is the Isolation Forest. It is a simple and efficient technique that isolates the anomalies at the upper levels of random trees (Liu, Ting & Zhou, 2008).

The method used to split the dataset into training and test subsets can introduce some bias in the pipeline. The distribution of the examples may not be similar in those two subsets, especially in small datasets (Hawkins, 2004). By evaluating the models several times using different and random train and test sets the prediction quality measurements could be more precise. In this case, $k$-fold cross-validation can be used, which splits the dataset into $k$ subsets. One fold is used to test the models and the remaining for training. In $k$ steps, folds are alternated. The average of the metrics in the test set is our final prediction quality measure for this model (Kuhn & Johnson, 2013).

## Proposed pipeline and experiments

**Figure 2:**
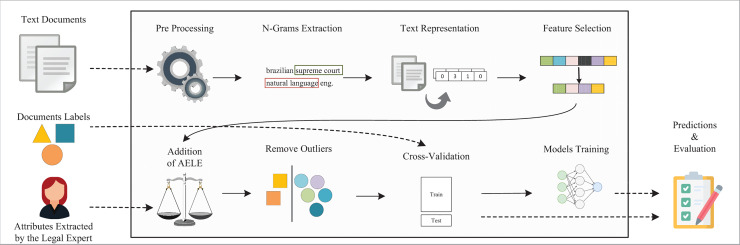
Full pipeline for regression in legal cases.

To answer the research question, we propose the application of several NLP and ML techniques on a pipeline for regression on legal texts (code available at https://github.com/thiagordp/text_regression_in_law_judgments). Thereby, we aim to create learning models capable of making accurate and helpful predictions for immaterial damage compensation. Figure 2 shows the proposed pipeline, built upon the one from “Regression applied to text data”. We incremented it with NLP and ML techniques, called adjustments, as we faced the challenges described in that section.

The pipeline receives three types of input: the text of the legal judgments, their labels, and the attributes extracted by a legal expert for each document (cf. “Dataset context and construction”). The pre-processing step converts the text to lowercase and remove noise characters, punctuation, stopwords such as *de*, *para* (prepositions in Portuguese), using the Natural Language Tool Kit (NLTK) (Bird & Loper, 2004).

The first adjustment is *N-Grams Extraction*, varying in length from one to four. However, as this range would lead to an unreasonable dimensionality, we limited the BOW representation to the 25,000 most frequent units, using Scikit-Learn (Pedregosa et al., 2012).

For the text representation, we use Bag of Words using term frequency (TF) values. We also tested word embeddings trained with legal documents written in Portuguese (Dal Pont et al., 2020), MultiLingual Bidirectional Encoder Representations from Transformer (BERT) (Devlin et al., 2018), and TF combined with Inverse Document Frequency (IDF), although TF achieved the best results for the experiments presented in this work. As an example, Multilingual BERT achieved a MAE of 6,192, a RMSE of 7,091, and a ${\rm R^2}$ of $- 3.21$. One hypothesis for this behavior is the size of our dataset that it is not enough for complex models, such as word embeddings and BERT, to represent and learn the continuous relationship between the texts and the compensation values. Furthermore, a similar situation occurs in the literature (Yeung, 2019), where frequency-based representations outperform BERT in the text regression task.

The second adjustment is *Feature Selection*, using the Mutual Information method. It maps the relationship between each feature (unit in the BOW) and the dependent variable (Cover & Thomas, 2005), the amount of immaterial damage compensation. As we tested a wide range of values as the number of features to select, we set it to 500 to consume less time on the experiments and still achieve good results.

The third adjustment is *Addition of AELE*, as described in “Dataset context and construction”. Categorical features such as *judges* and *types of judges* were converted to one-hot encoding. Real value features, such as *delay interval*, were not modified. In the end, the final representations of the documents were composed of 52 features from the legal attributes and the BOW features. That is, 500 when feature selection is activated or 50,000, otherwise.

The fourth adjustment is *Outliers Removal*. As previously described, outliers are very distinctive examples in the dataset, and by removing them, we make it easier for the models to learn. To detect outliers, we used the Isolation Forest with contamination set to ten percent. Moreover, we have placed this step in two different positions: before and after cross-validation, but we did not apply both in the same pipeline. The former intends to remove outliers from the whole dataset, while the latter, from the train set. By removing outliers from all dataset, we imply that our future cases for prediction will not contain outliers.

The fifth adjustment is *Cross-Validation*, which uses multiple combinations of the train and test sets and the resulting metrics will be averaged. In this work, we set the number of folds to five, so, in each step, eighty percent and twenty percent of the dataset is used for train and test, respectively.

The selected techniques of ML for the regression task are listed in Table 1, where the parameters values were defined empirically based on a series of previous experiments. Considering the problem of overfitting, we evaluate the techniques for two configurations: *simple* and *complex*. In the former, we define some constraints to the models such as the number of iterations and maximum tree levels, while in the latter we let the models free, without such constraints. The fourth column of the table contains the parameter values used in both configurations and any unlisted parameters in Table 1 follow the default values from Scikit-Learn (Pedregosa et al., 2012).

**Table 1:** Regression techniques and parameters.

Technique	Parameters (complex)	Parameters (simple)	Common parameters
AdaBoost	N° estimators: 100	N° estimators: 50	Learning rate: 0.1
Bagging	N° estimators: 100	N° estimators: 50	–
Decision tree	Maximum depth: unlimited	Maximum depth: 10	–
	Max leaf nodes: unlimited	Max leaf nodes: 100	
Neural network	Hidden layers: 5	Hidden layers: 5	Activation: ReLU
	Neurons: 512 (each layer)	Neurons: 256 (each layer)	Batch size: 16
	Max iterations: 100	Max iterations: 50	
	Early stopping: deactivated	Early stopping: actived	
Elastic net	Max iterations: 100	Max iterations: 50	–
Ensemble voting	Bagging	Bagging	–
	Neural network	Neural network	–
	Gradient boosting	Gradient boosting	–
	XGBoosting	XGBoosting	–
Gradient boosting	N° estimators: 100	N° estimators: 50	–
	Max depth: unlimited	Max depth: 10	–
	Max leaf nodes: unlimited	Max leaf nodes: 100	–
Random forest	N° estimators: 100	N° estimators: 50	–
	Max depth: unlimited	Max depth: 10	–
	Max leaf nodes: unlimited	Max leaf nodes: 100	–
Ridge	Max iterations: 100	Max iterations: 50	Alpha: 0.1
			Tolerance: 0.001
SVM	Max iterations: 100	Max iterations: 50	C: 1.0
			Epsilon: 0.2
			Kernel: RBF
XGBoosting	N° estimators: 100	N° estimators: 50	–
	Max depth: unlimited	Max depth: 10	–

Finally, the sixth adjustment is *Overfitting Avoidance*, which is implemented by simpler models in our pipeline. We note that Ensemble Voting Model is an ensemble of ensembles, so it uses models like Bagging and XGBoosting with the same parameters as described in their respective lines.

The final step, as described in “Regression applied to text data”, is the evaluation of our models. From their predictions on the test set, we measure the prediction quality using three metrics: Root Mean Square Error (RMSE), Mean Absolute Error (MAE), and the Coefficient of Determination ( ${\rm R^2}$).

In the experimental setup, we initially evaluate the two pipelines of Figs. 1 and 2, which we call *baseline* and *full* pipelines, respectively. Thereby, we can have an overall estimate of how much our adjustments in the *full* pipeline improve the regression metrics.

Furthermore, to verify in what extent the prediction is accurate, we also performed some experiments with other combinations of adjustments, for instance, bypassing *N-Grams Extraction* and *Feature Selection*, while keeping *Addition of AELE*, *Outliers Removal*, *Cross-Validation*, and *Overfitting Avoidance*. With the experiments for the different pipelines, we can also measure how much each adjustment contributes for the performance of the models.

To run the experiments, we first set which adjustments to use, that in total embraced 80 combinations. For each combination, we executed the pipeline twenty-five times. In each repetition, if *Cross-Validation* is disabled, we only train and test the models once, and we do it five times, otherwise. To get the final metrics of the set of repetitions, we took the average for MAE, RMSE, and ${\rm R^2}$ among the repetitions.

## Results and discussion

This section presents the results from the experiments regarding the different pipelines: *baseline*, *full*, and the 80 combinations of adjustments. We analyze the adjustments’ influence in terms of prediction quality and execution time.

### Results from baseline and full pipelines

**Figure 3:**
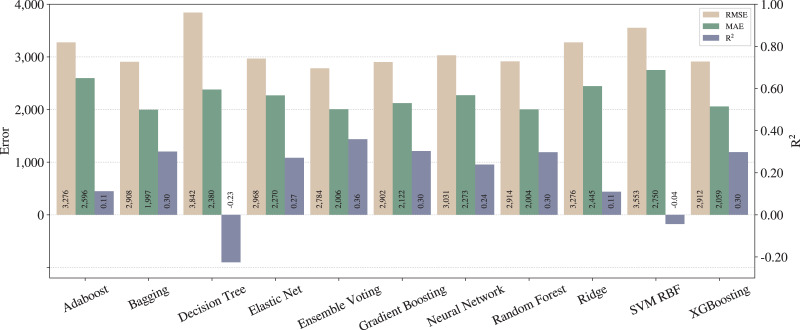
Results from baseline pipeline.

Considering the steps described in “Proposed pipeline and experiments”, we run the experiments for the *baseline* as shown in Fig. 1. This setup does not include the adjustments. Figure 3 presents the results for each regression model, in which the left $y$-axis relates to errors (RMSE and MAE) while the right $y$-axis to ${\rm R^2}$.

**Figure 4:**
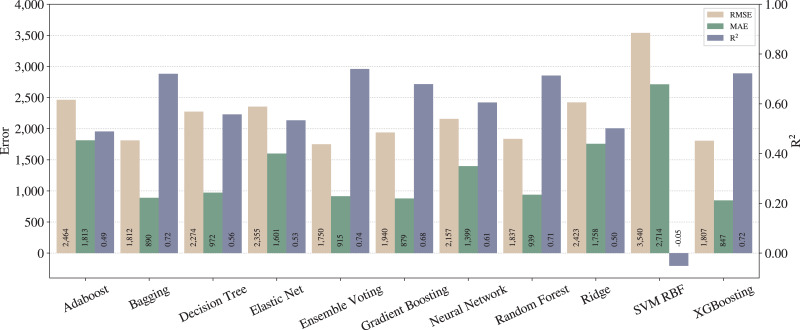
Results from full pipeline.

We repeated the steps of the *full pipeline* from “Proposed pipeline and experiments” with all adjustments activated (except outliers removal in training data) and the results are shown in Fig. 4.

From Figs. 3 and 4, one can notice significant improvements on the three metrics for most of the techniques, except for SVM with RBF kernel. In that case, we can infer that SVM is underfitted, since the poor results stood regardless the applied pipelines. On the other hand, in terms of the best techniques, we can realize that Ensemble Voting achieves the best results among the techniques in terms of RMSE and ${\rm R^2}$. Thus, merging the techniques in Ensemble Voting achieves better results when compared to the models alone for ${\rm R^2}$ and RMSE. XGBoosting produces the best prediction quality in terms of MAE.

As described in “Regression applied to text data”, RMSE tends to penalize bigger errors, while MAE does not, so we can state that Ensemble Voting has fewer large errors than XGBoosting. Still, it predicts incorrectly more examples than XGBoosting.

As expected, we can conclude that the *full* pipeline leads to better results than *baseline*. Moreover, from the legal expert experience, an ${\rm R^2}$ of 0.74 can be considered as a good amount for this metric, and also an MAE of less than one thousand can be considered almost irrelevant in the context of legal compensation.

### Results from combinations of adjustments

This section presents the performance of combinations of adjustments and whether they achieve any better result when compared to the *full* pipeline. Considering again Fig. 2, we randomly selected a total of eighty different pipelines. For instance, we kept *N-Grams Extraction*, *Addition of AELE* and *Cross-Validation* bypassing *Feature Selection*, and *Outliers Removal*. When we bypass an adjustment, we connect its predecessor step in the pipeline with its successor. For example, if we bypass *N-Grams Extraction*, the pre-processing step will be connected to the representation step and so on. Furthermore, the pipeline stays the same despite the (de)activation of *Overfitting Avoidance* adjustment. This adjustment is more related to the configuration for the training step, that is, use complex (when deactivated) or simpler models (when activated) from Table 1.

We represent a combination of adjustments as a binary number. If the adjustment is bypassed the digit is zero, and it is one otherwise. We assigned positions in the binary number to adjustments in this order, from left to right: *Feature Selection*, *Outlier Removal (Train Set)*, *N-Grams Extraction*, *Addition of AELE*, *Cross-Validation*, *Overfitting Avoidance* and *Outlier Removal (All Dataset)*.

**Figure 5:**
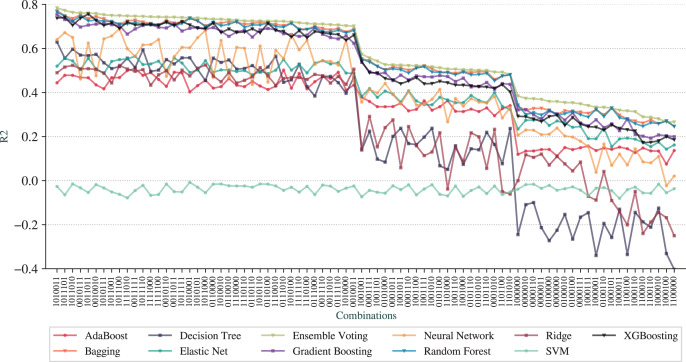
${\rm R^2}$ for the pipelines based on combinations of adjustments.

Figure 5 shows the results, in which the $x$-axis represents the combinations, the $y$-axis represents the ${\rm R^2}$ metric and each line is a different technique. To better detect the patterns, we have arranged the combinations in decreasing order of ${\rm R^2}$ from Ensemble Voting regression.

**Figure 6:**
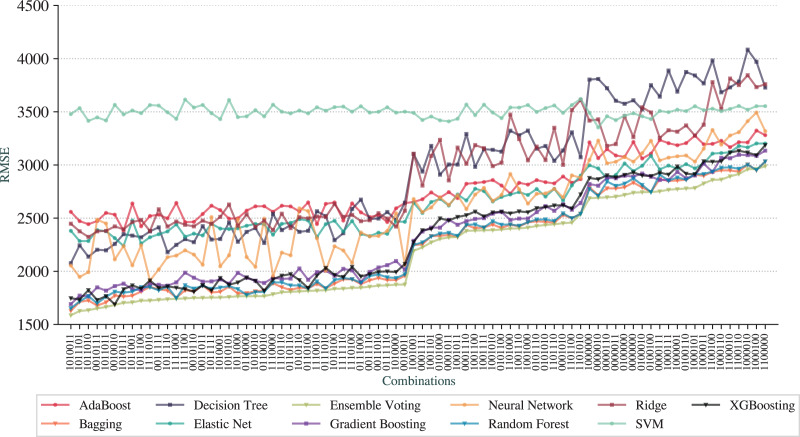
RMSE for the pipelines based on combinations of adjustments.

Following the same idea, Fig. 6 shows the results for RMSE draw from the same order of combinations in the $x$-axis.

The first observation is that we can achieve prediction qualities better than the *full* pipeline. The best pipeline is represented as $1010011$, with *Feature Selection*, *N-Grams Extraction*, *Overfitting Avoidance* and *Outlier Removal (All Dataset)* activated while *Outlier Removal (Train Set)*, *Addition of AELE* and *Cross-Validation* deactivated. The best technique is the Ensemble Voting with ${\rm R^2}$ of 0.78, RMSE of 1,586, and MAE of 803. The results for combinations for MAE are not included due to the similarity to RMSE results.

We can observe that Ensemble Voting, Bagging, Random Forest, Gradient Boosting, and XGBoosting have the best results as they stand at the top of the charts for most combinations. As the worse technique, we have SVM, that performed poorer than other techniques in most combinations. Another observation is that the *baseline* pipeline is better than 20 combinations in terms of prediction quality.

From a more global analysis of Figs. 5 and 6, there are two sudden changes in the prediction quality. The first happens in the middle of the graphs for RMSE and ${\rm R^2}$ and the second appears in the third quarter. We can notice that most of the techniques exhibit this behavior of sudden change. The first change happens when the combinations no longer contain *N-Grams Extraction* (third digit in the digital notation) as, before that point, all combinations have this adjustment. We can also note that *Addition of AELE* (fourth digit in the digital notation) starts to appear consistently in all combinations of adjustments from this point.

The second sudden change happens when *Addition of AELE* stops appearing in the combinations. As we described, the best combination does not have this adjustment, but at this point, it has a clear impact on the prediction quality. Thus, we can notice that the cause of this difference is the presence of *N-Grams Extraction*. With *N-Grams Extraction*, the impact of *Addition of AELE* on the prediction quality reduces and it increases, otherwise. This pattern is shared among all models except SVM, which did not perform well in any combinations.

Although different combinations of adjustments can lead to results better than the full pipeline, it is important to quantify how much each adjustment impacts the results. As demonstrated, *N-Grams Extraction* and *Addition of AELE* have a considerable impact on the prediction quality.

### Impact of each adjustment on the performance

This section discusses how each adjustment impacts on the prediction quality and execution time. We investigate how much RMSE, ${\rm R^2}$, and execution time varies as we add or remove steps in the pipeline. To do so, for each adjustment, we selected the results from all the pipelines where the adjustment is bypassed, then also selected the results from the pipelines where it is activated, forming two sets. With the aim of testing whether the two sets has a statistically significant difference, that is, whether the adjustments have a real impact on the results, we performed unpaired one-tailed Mann–Whitney U hypothesis tests, with 95% confidence ( $\alpha$ = 0.05). The sets come from different pipelines, thus they are unpaired.

For the RMSE results, the null hypothesis ( ${H_0}$) indicates that RMSE results *remain the same or increase* in pipelines with the adjustment, and the alternative hypothesis ( ${H_a}$) indicates that RMSE results *decrease* in pipelines with the adjustment. For ${\rm R^2}$ results, ${H_0}$ indicates that ${\rm R^2}$ results *remain the same or decrease* in pipelines with the adjustment, and ${H_a}$ indicates the ${\rm R^2}$ *increase* in pipelines with the adjustment.

The results for RMSE and ${\rm R^2}$ are briefly presented in Tables 2 and 3, respectively, and they are detailed in the Appendix. There are two medians, *Median 1* and *Median 2*, indicating central tendency of each of the two sets with and without adjustments, respectively.

**Table 2:** Medians of RMSE measures for each regression technique in pipelines with and without each adjustment (Median 1 and 2, respectively) and the resulting *p*-values for unpaired one-tailed Mann–Whitney U test for RMSE with 95% confidence (bold values indicate statistical significance. For more details, please check the Table B1 in the Appendix section). ${H_0}$: RMSE measures remain the same or increase in pipelines with the adjustment. ${H_a}$: RMSE measures decrease in pipelines with the adjustment.

Tech	Stat	Feature selection	Addition of AELE	Cross-validation	N-grams extraction	Overfitting avoidance	Outliers removal (train)	Outliers removal (all)
Adaboost	Median 1	2,650	2,652	2,677	2,549	2,663	2,730	2,648
	Median 2	2,711	3,060	2,652	3,060	2,691	2,652	2,769
	*p*-value	0.51	**<0.001**	0.54	**<0.001**	0.15	0.56	0.09
Bagging	Median 1	2,102	2,246	2,254	1,824	2,096	2,407	2,082
	Median 2	2,297	2,716	2,246	2,716	2,288	2,246	2,405
	*p*-value	0.38	0.06	0.56	**<0.001**	0.28	0.91	**0.02**
Decision tree	Median 1	2,777	2,909	2,938	2,371	2,737	3,039	2,696
	Median 2	2,971	3,522	2,909	3,522	3,126	2,909	3,010
	*p*-value	0.32	**0.04**	0.58	**<0.001**	**0.03**	0.87	0.07
Elastic net	Median 1	2,561	2,548	2,548	2,381	2,519	2,666	2,513
	Median 2	2,583	2,872	2,618	2,900	2,648	2,548	2,661
	*p*-value	0.70	**<0.001**	0.69	**<0.001**	0.28	0.80	0.14
Ensemble voting	Median 1	2,034	2,193	2,225	1,765	2,050	2,381	2,034
	Median 2	2,272	2,687	2,193	2,687	2,233	2,193	2,382
	*p*-value	0.28	**0.01**	0.56	**<0.001**	0.31	0.93	**0.02**
Gradient boosting	Median 1	2,155	2,287	2,374	1,920	2,235	2,471	2,191
	Median 2	2,422	2,805	2,287	2,805	2,349	2,287	2,477
	*p*-value	0.32	**0.01**	0.62	**<0.001**	0.58	0.90	**0.04**
Neural network	Median 1	2,418	2,557	2,592	2,157	2,574	2,592	2,551
	Median 2	2,588	3,016	2,585	3,016	2,593	2,557	2,614
	*p*-value	0.10	**0.01**	0.50	**<0.001**	0.63	0.90	0.16
Random forest	Median 1	2,110	2,247	2,272	1,851	2,121	2,437	2,092
	Median 2	2,317	2,710	2,247	2,710	2,288	2,247	2,420
	*p*-value	0.32	0.06	0.53	**<0.001**	0.56	0.90	**0.02**
Ridge	Median 1	2,860	2,806	2,806	2,471	2,720	3,012	2,695
	Median 2	2,830	3,178	2,854	3,276	3,028	2,806	2,995
	*p*-value	0.89	**0.04**	0.31	**<0.001**	0.18	0.96	0.09
SVM (RBF)	Median 1	3,514	3,540	3,507	3,501	3,493	3,535	3,462
	Median 2	3,492	3,497	3,497	3,506	3,524	3,490	3,514
	*p*-value	0.97	>0.99	0.74	0.62	**0.01**	>0.99	**<0.001**
XGBoosting	Median 1	2,172	2,274	2,386	1,895	2,192	2,527	2,134
	Median 2	2,432	2,866	2,274	2,866	2,336	2,274	2,522
	*p*-value	0.45	0.01	0.56	**<0.001**	0.41	0.87	**0.02**

**Table 3:** Medians of ${\rm R^2}$ measures for each regression technique in pipelines with and without each adjustment and the resulting *p*-values for unpaired one-tailed Mann–Whitney U test for ${\rm R^2}$ with 95% confidence (bold values indicate statistical significance. For more details, please check the Table B2 in the Appendix section). ${H_0}$: ${\rm R^2}$ measures remain the same or decrease in pipelines with the adjustment. ${H_a}$: ${\rm R^2}$ measures increase in pipelines with the adjustment.

Tech	Stat	Feature selection	Addition of AELE	Cross-validation	N-grams extraction	Overfitting avoidance	Outliers removal (train)	Outliers removal (all)
Adaboost	Median 1	0.39	0.38	0.36	0.46	0.39	0.36	0.39
	Median 2	0.35	0.16	0.38	0.16	0.37	0.38	0.35
	*p*-value	0.50	**<0.001**	0.48	**<0.001**	0.13	0.39	0.15
Bagging	Median 1	0.61	0.55	0.55	0.72	0.61	0.50	0.62
	Median 2	0.53	0.34	0.55	0.34	0.54	0.55	0.51
	*p*-value	0.37	0.05	0.50	**<0.001**	0.31	0.91	**0.03**
Decision tree	Median 1	0.32	0.24	0.22	0.52	0.34	0.24	0.35
	Median 2	0.21	−0.10	0.24	−0.10	0.15	0.24	0.24
	*p*-value	0.32	**0.04**	0.56	**< 0.001**	**0.02**	0.82	0.16
Elastic net	Median 1	0.43	0.42	0.42	0.52	0.45	0.41	0.45
	Median 2	0.41	0.28	0.41	0.28	0.39	0.42	0.41
	*p*-value	0.74	**<0.001**	0.63	**<0.001**	0.26	0.70	0.28
Ensemble voting	Median 1	0.64	0.57	0.56	0.73	0.63	0.53	0.64
	Median 2	0.54	0.38	0.57	0.38	0.56	0.57	0.52
	*p*-value	0.34	**0.01**	0.55	**<0.001**	0.38	0.89	0.06
Gradient boosting	Median 1	0.60	0.54	0.49	0.69	0.56	0.49	0.58
	Median 2	0.49	0.32	0.54	0.32	0.51	0.54	0.48
	*p*-value	0.30	**0.01**	0.59	**<0.001**	0.69	0.85	0.10
Neural network	Median 1	0.49	0.44	0.41	0.60	0.42	0.44	0.43
	Median 2	0.43	0.24	0.44	0.24	0.43	0.41	0.43
	*p*-value	0.12	**0.01**	0.47	**<0.001**	0.65	0.84	0.32
Random forest	Median 1	0.61	0.55	0.54	0.71	0.61	0.50	0.62
	Median 2	0.52	0.34	0.55	0.34	0.54	0.55	0.51
	*p*-value	0.30	0.05	0.50	**<0.001**	0.64	0.86	**0.03**
Ridge	Median 1	0.30	0.29	0.29	0.48	0.36	0.24	0.37
	Median 2	0.28	0.12	0.28	0.08	0.23	0.29	0.24
	*p*-value	0.87	**0.03**	0.30	**<0.001**	0.20	0.93	0.19
SVM (RBF)	Median 1	−0.04	−0.05	−0.04	−0.03	−0.03	−0.04	−0.04
	Median 2	−0.03	−0.03	−0.04	−0.04	−0.05	−0.03	−0.03
	*p*-value	0.99	>0.99	0.72	**0.03**	**<0.001**	0.94	0.94
XGBoosting	Median 1	0.59	0.54	0.49	0.69	0.58	0.46	0.60
	Median 2	0.48	0.30	0.54	0.30	0.52	0.54	0.47
	*p*-value	0.53	**0.01**	0.53	**<0.001**	0.51	0.80	0.07

**Table B1:** Mann–Whitney U tests details of the differences for RMSE with 95% confidence (bold values indicate statistical significance). ${H_0}$: RMSE measures remain the same or increase in pipelines with the adjustment. ${H_a}$: RMSE measures decrease in pipelines with the adjustment.

Tech	Stat	Addition of AELE	Cross-validation	Feature selection	N-grams extraction	Outliers removal (all)	Outliers removal (train)	Overfitting avoidance
Adaboost	N1	39	39	48	39	24	31	48
	N2	41	41	32	41	56	49	32
	Median 1	2,652	2,677	2,650	2,549	2,648	2,730	2,663
	Median 2	3,060	2,652	2,711	3,060	2,769	2,652	2,691
	Stat	422	809	771	0	543	775	662
	*p*-value	**<0.001**	0.54	0.51	**<0.001**	0.09	0.56	0.15
Bagging	N1	39	39	48	39	24	31	48
	N2	41	41	32	41	56	49	32
	Median 1	2,246	2,254	2,102	1,824	2,082	2,407	2,096
	Median 2	2,716	2,246	2,297	2,716	2,405	2,246	2,288
	Stat	637	814	736	0	468	895	707
	*p*-value	0.06	0.56	0.38	**<0.001**	0.02	0.91	0.28
Decision tree	N1	39	39	48	39	24	31	48
	N2	41	41	32	41	56	49	32
	Median 1	2,909	2,938	2,777	2,371	2,696	3,039	2,737
	Median 2	3,522	2,909	2,971	3,522	3,010	2,909	3,126
	Stat	617	819	720	0	532	872	570
	*p*-value	**0.04**	0.58	0.32	**<0.001**	0.07	0.87	**0.03**
Elastic net	N1	39	39	48	39	24	31	48
	N2	41	41	32	41	56	49	32
	Median 1	2,548	2,548	2,561	2,381	2,513	2,666	2,519
	Median 2	2,872	2,618	2,583	2,900	2,661	2,548	2,648
	Stat	404	850	822	0	567	845	708
	*p*-value	**<0.001**	0.69	0.70	**<0.001**	0.14	0.80	0.28
Ensemble voting	N1	39	39	48	39	24	31	48
	N2	41	41	32	41	56	49	32
	Median 1	2,193	2,225	2,034	1,765	2,034	2,381	2,050
	Median 2	2,687	2,193	2,272	2,687	2,382	2,193	2,233
	Stat	561	815	707	0	474	909	718
	*p*-value	**0.01**	0.56	0.28	**<0.001**	**0.02**	0.93	0.31
Gradient boosting	N1	39	39	48	39	24	31	48
	N2	41	41	32	41	56	49	32
	Median 1	2,287	2,374	2,155	1,920	2,191	2,471	2,235
	Median 2	2,805	2,287	2,422	2,805	2,477	2,287	2,349
	Stat	566	831	720	0	506	886	787
	*p*-value	**0.01**	0.62	0.32	**<0.001**	**0.04**	0.90	0.58
Neural network	N1	39	39	48	39	24	31	48
	N2	41	41	32	41	56	49	32
	Median 1	2,557	2,592	2,418	2,157	2,551	2,592	2,574
	Median 2	3,016	2,585	2,588	3,016	2,614	2,557	2,593
	Stat	544	800	636	3	576	889	802
	*p*-value	**0.01**	0.50	0.10	**<0.001**	0.16	0.90	0.63
Random forest	N1	39	39	48	39	24	31	48
	N2	41	41	32	41	56	49	32
	Median 1	2,247	2,272	2,110	1,851	2,092	2,437	2,121
	Median 2	2,710	2,247	2,317	2,710	2,420	2,247	2,288
	Stat	639	808	721	0	467	886	784
	*p*-value	0.06	0.53	0.32	**<0.001**	**0.02**	0.90	0.56
Ridge	N1	39	39	48	39	24	31	48
	N2	41	41	32	41	56	49	32
	Median 1	2,806	2,806	2,860	2,471	2,695	3,012	2,720
	Median 2	3,178	2,854	2,830	3,276	2,995	2,806	3,028
	Stat	617	748	892	0	542	934	674
	*p*-value	**0.04**	0.31	0.89	**<0.001**	0.09	0.96	0.18
SVM (RBF)	N1	39	39	48	39	24	31	48
	N2	41	41	32	41	56	49	32
	Median 1	3,540	3,507	3,514	3,501	3,462	3,535	3,493
	Median 2	3,497	3,497	3,492	3,506	3,514	3,490	3,524
	Stat	1,068	866	958	831	319	1,131	534
	*p*-value	>0.99	0.74	0.97	0.62	**<0.001**	>0.99	**0.01**
XGBoosting	N1	39	39	48	39	24	31	48
	N2	41	41	32	41	56	49	32
	Median 1	2,274	2,386	2,172	1,895	2,134	2,527	2,192
	Median 2	2,866	2,274	2,432	2,866	2,522	2,274	2,336
	Stat	564	814	755	0	474	871	745
	*p*-value	**0.01**	0.56	0.45	**<0.001**	**0.02**	0.87	0.41

**Table B2:** Mann–Whitney U tests details of the differences for ${\rm R^2}$ with 95% confidence (bold values indicate statistical significance). ${H_0}$: ${\rm R^2}$ measures remain the same or decrease in pipelines with the adjustment. ${H_a}$: ${\rm R^2}$ measures increase in pipelines with the adjustment.

Tech	Stat	Addition of AELE	Cross-validation	Feature selection	N-grams extraction	Outliers removal (all)	Outliers removal (train)	Overfitting avoidance
Adaboost	N1	39	39	48	39	24	31	48
	N2	41	41	32	41	56	49	32
	Median 1	0.38	0.36	0.39	0.46	0.39	0.36	0.39
	Median 2	0.16	0.38	0.35	0.16	0.35	0.38	0.37
	Stat	1,188	806	769	1,599	772	789	884
	*p*-value	**<0.001**	0.48	0.50	**<0.001**	0.15	0.39	0.13
Bagging	N1	39	39	48	39	24	31	48
	N2	41	41	32	41	56	49	32
	Median 1	0.55	0.55	0.61	0.72	0.62	0.50	0.61
	Median 2	0.34	0.55	0.53	0.34	0.51	0.55	0.54
	Stat	976	801	801	1,599	858	625	820
	*p*-value	0.05	0.50	0.37	**<0.001**	**0.03**	0.91	0.31
Decision tree	N1	39	39	48	39	24	31	48
	N2	41	41	32	41	56	49	32
	Median 1	0.24	0.22	0.32	0.52	0.35	0.24	0.34
	Median 2	−0.10	0.24	0.21	−0.10	0.24	0.24	0.15
	Stat	985	783	816	1,599	766	666	979
	*p*-value	**0.04**	0.56	0.32	**<0.001**	0.16	0.82	0.02
Elastic net	N1	39	39	48	39	24	31	48
	N2	41	41	32	41	56	49	32
	Median 1	0.42	0.42	0.43	0.52	0.45	0.41	0.45
	Median 2	0.28	0.41	0.41	0.28	0.41	0.42	0.39
	Stat	1,199	766	704	1,599	728	708	835
	*p*-value	**<0.001**	0.63	0.74	**<0.001**	0.28	0.70	0.26
Ensemble voting	N1	39	39	48	39	24	31	48
	N2	41	41	32	41	56	49	32
	Median 1	0.57	0.56	0.64	0.73	0.64	0.53	0.63
	Median 2	0.38	0.57	0.54	0.38	0.52	0.57	0.56
	Stat	1,045	787	810	1,599	822	635	799
	*p*-value	**0.01**	0.55	0.34	**<0.001**	0.06	0.89	0.38
Gradient boosting	N1	39	39	48	39	24	31	48
	N2	41	41	32	41	56	49	32
	Median 1	0.54	0.49	0.60	0.69	0.58	0.49	0.56
	Median 2	0.32	0.54	0.49	0.32	0.48	0.54	0.51
	Stat	1,042	776	821	1,599	796	657	718
	*p*-value	**0.01**	0.59	0.30	**<0.001**	0.10	0.85	0.69
Neural network	N1	39	39	48	39	24	31	48
	N2	41	41	32	41	56	49	32
	Median 1	0.44	0.41	0.49	0.60	0.43	0.44	0.42
	Median 2	0.24	0.44	0.43	0.24	0.43	0.41	0.43
	Stat	1,060	809	888	1,598	717	660	729
	*p*-value	**0.01**	0.47	0.12	**<0.001**	0.32	0.84	0.65
Random forest	N1	39	39	48	39	24	31	48
	N2	41	41	32	41	56	49	32
	Median 1	0.55	0.54	0.61	0.71	0.62	0.50	0.61
	Median 2	0.34	0.55	0.52	0.34	0.51	0.55	0.54
	Stat	969	801	822	1,599	847	649	732
	*p*-value	0.05	0.50	0.30	**<0.001**	**0.03**	0.86	0.64
Ridge	N1	39	39	48	39	24	31	48
	N2	41	41	32	41	56	49	32
	Median 1	0.29	0.29	0.30	0.48	0.37	0.24	0.36
	Median 2	0.12	0.28	0.28	0.08	0.24	0.29	0.23
	Stat	991	855	655	1,599	757	611	853
	*p*-value	**0.03**	0.30	0.87	**<0.001**	0.19	0.93	0.20
SVM (RBF)	N1	39	39	48	39	24	31	48
	N2	41	41	32	41	56	49	32
	Median 1	−0.05	−0.04	−0.04	−0.03	−0.04	−0.04	−0.03
	Median 2	−0.03	−0.04	−0.03	−0.04	−0.03	−0.03	−0.05
	Stat	402	740	508	995	525	602	1,040
	*p*-value	>0.99	0.72	0.99	**0.03**	0.94	0.94	**<0.001**
XGBoosting	N1	39	39	48	39	24	31	48
	N2	41	41	32	41	56	49	32
	Median 1	0.54	0.49	0.59	0.69	0.60	0.46	0.58
	Median 2	0.30	0.54	0.48	0.30	0.47	0.54	0.52
	Stat	1,047	792	761	1,599	816	674	766
	*p*-value	**0.01**	0.53	0.53	**<0.001**	0.07	0.80	0.51

Furthermore, one distinct hypothesis test was performed for each pair of regression technique and adjustment, and the corresponding *p*-value is presented. As an example, from Table 2, the statistical test to check whether *Addition of AELE* has an impact on RMSE from *Adaboost* resulted in a *p*-value *< 0.001* (which is lower than the $\alpha$), and thus, it is statistically significant. Finally, we **highlighted** the p-values which indicate the statistical significant difference between the two results sets, that is, *p*-values lower than the $\alpha$.

From Tables 2 and 3, we confirm the observations from the previous sections about the sudden changes in which the combinations do not have *N-Grams Extraction* or *Addition of AELE*. Here, we measure the impact of these two adjustments on the prediction quality, as the two sets (represented by Median 1 and Median 2) are significantly different according to the *p*-values.

In terms of regression techniques, when comparing the medians, the adjustments have a significant impact on the Decision Tree and least impact on SVM. Tree-based methods, such as XGBoosting, Random Forest and Bagging, also performed significantly better with the application of the adjustments.

Tables 2 and 3 also show that *Feature Selection*, *Cross-Validation* and *Outliers Removal in train set* have no significant impact. However, *Outliers Removal from all the dataset* significantly impacts on half of the techniques results for RMSE.

Regarding the *execution time*, Table 4 contains the results. It presents the medians of execution time (in hours) of the whole pipeline for the sets with (Median 1) and without the adjustment (Median 2). Even though *Feature Selection* has no significant impact on prediction quality, when it is activated, the execution time decreases. Something similar happens to *Overfitting Avoidance* since it also has little impact, but the execution time reduces significantly. Therefore, there is a trade-off between execution time and prediction quality we have to balance. It pays off to have *Feature Selection* and *Overfitting Avoidance* adjustments in our pipeline, since when we have both bypassed executing the whole pipeline took hours in our experiments, while it took half of the time in the opposite situation.

**Table 4:** Resulting statistics for *p*-values for unpaired one-tailed Mann–Whitney U test for execution time with 95% confidence (bold values indicate statistical significance. For more details, please check the Table B3 in the Appendix section). ${H_0}$: execution time remains the same or increase in pipelines with the adjustment. ${H_a}$: execution time reduces in pipelines with the adjustment.

Stats	Feature selection	Addition of AELE	Cross-validation	N-grams extraction	Overfitting avoidance	Outliers removal (train)	Outliers removal (all)
Median 1	0.80	2.27	6.32	3.10	1.50	3.44	1.62
Median 2	7.51	2.20	1.32	2.20	3.56	1.91	2.95
*p*-value	**<0.001**	0.87	>0.99	0.65	**0.02**	0.85	0.18

**Table B3:** Resulting statistics for *p*-values for unpaired one-tailed Mann–Whitney U test for execution time with 95% confidence (bold values indicate statistical significance). ${H_0}$: execution time remains the same or increase in pipelines with the adjustment. ${H_a}$: execution time reduces in pipelines with the adjustment.

Stats	Feature selection	Addition of AELE	Cross-validation	N-grams extraction	Overfitting avoidance	Outliers removal (train)	Outliers removal (all)
N1	48	39	39	39	48	31	24
N2	32	41	41	41	32	49	56
Median 1	0.80	2.27	6.32	3.10	1.50	3.44	1.62
Median 2	7.51	2.20	1.32	2.20	3.56	1.91	2.95
Stat	136	918	1,225	839	560	862.5	582.5
*p*-value	**<0.001**	0.87	>0.99	0.65	**0.02**	0.85	0.18

Although we can see that *Cross-Validation* also has no impact on the prediction quality in the regression task, it increases the execution time almost five times. As presented in “Regression applied to text data”, cross-validation tends to produce results with less bias since it generates five different combinations to train and test our models. But, in this experiment, we do not notice this effect. The twenty five repetitions we run without *Cross-Validation* produced different combinations of train and test sets with a good amount of variability to capture a good estimation of the model prediction quality. Thus, the five-fold extra combinations from cross-validation do not impact the results significantly in our experiments. Except for the time execution, which took almost five times longer.

*Outliers Removal* also do not impact significantly the results. But we can see that the two approaches, that is, removing outliers in the train data or the whole dataset, influence the results in different ways. While the former tends to lead to worse results, the latter leads to better results. When we remove outliers from train data and keep them in the test data, the models make poor predictions for the outliers but, if we keep our entire dataset away from anomalies, the models get better prediction quality results.

We also note that *N-Grams Extraction* and *Addition of AELE*, in terms of execution time, tend to impact negatively the pipeline, as shown in Table 4. Still, the improvement in the pipeline results overlaps this additional execution time when considering the high gains in prediction quality on adding these adjustments.

Finally, considering prediction quality and execution time, the best combination is $1011011$, with the adjustments *Feature Selection*, *N-Grams Extraction*, *Addition of AELE*, *Overfitting Avoidance* and *Outliers Removal* from all dataset activated and the remaining deactivated. This is the fifth combination from Figs. 5 and 6. In terms of prediction quality, the best model, Ensemble Voting achieved a RMSE of 1,683, a MAE of 866 and ${\rm R^2}$ of 0.76.

## Conclusions and future work

This article presents the results of an investigation about the application of text regression techniques to predict the compensation value for immaterial damage. In the first part, we evaluated two pipelines, which we called *baseline* and *full* pipelines. The former is the simplest pipeline for regression in text and the latter is based on the former with some improvements that we call *adjustments*. By testing several regression techniques, we confirmed that the *full* pipeline achieves superior results. The best technique in these experiments was Ensemble Voting with an ${\rm R^2}$ of 0.74, RMSE of 1,750 and MAE of 915.

In the second part of this article, we further evaluate the proposed adjustments, that is, *Feature Selection*, *Cross-Validation*, *Addition of AELE*, *N-Grams Extraction*, *Overfitting Avoidance*, and *Outliers Removal*. We tested 80 distinct combinations of pipelines and highlighted some combinations of adjustments that achieved better prediction quality than the *full* pipeline, that is, combinations with fewer steps in the pipeline.

Answering the research question, we can conclude that the best pipeline has accurate predictions for the application text regression. From the considered adjustments, *N-Grams Extraction* and *Addition of AELE* produce gains on the prediction quality while slightly increasing execution time. When using *Feature Selection* and *Overfitting Avoidance* the execution time reduced considerably. Thus, adopting these four adjustments in the pipelines implies in gains in terms of prediction quality and execution time.

Also, the evaluation of the results from a legal expert’s perspective shows that the predictions are helpful in the legal environment and can encourage the parties involved (consumer and airline) in an agreement. The MAE error of the best pipeline was 866.00. That way, giving up approximately 1,000 Brazilian Reais of the compensation is acceptable in conciliation hearings (an initial lawsuit stage in which the parties try to negotiate to solve the case themselves). For example, the consumer who will earn R$ 5,000 only at the end of the lawsuit, will agree more easily to being compensated in R$ 4,000 in the beginning, so the case is closed immediately. By obtaining more agreements, a positive impact on the Justice response time is achieved.

In terms of future work, we intend to apply the models in a real context of the Special State Court and verify in how many cases we can help to finish in the conciliation hearing. Other legal contexts such as criminal and administrative can be addressed. Deep learning techniques may be applied and tested in these applications, although we could not get good results using these techniques at the early steps of this research. Improvements in the legal text representation will be addressed using pre-trained models for Portuguese such as BERTimbau, verBERT, and others (Souza, Nogueira & Lotufo, 2020; Serras & Finger, 2022).

### Results from the statistical tests

In this section, we present the details of the Mann–Whitney U-tests with 95% confidence. Therefore, in the following tables, we present the test statistics, sizes and medians of each sample and, finally, the $p$-value. ${H_0}$: RMSE measures remain the same or increase in pipelines with the adjustment. ${H_a}$: RMSE measures decrease in pipelines with the adjustment.

## Appendix

### Details on data anonymization

In this section, we present the details regarding the anonymization of the dataset used in the experimentation part of the article. Considering the experimentation scope, the data removed was not relevant to the experiments.

As the documents have personal information regarding the parties in the process (their names), it was required to remove their information from the text. The process identification was also removed to avoid indirect identification.

**Figure A1:**
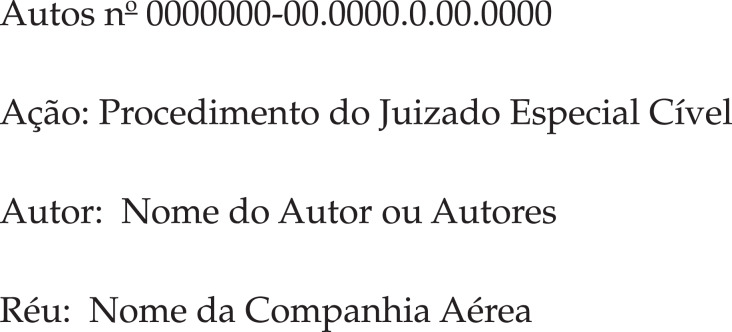
Legal Case header example

In the legal documents, the identification of the process and the parties is done in the header of the process as exemplified in Fig. A1.

From Fig. A1, one can see the process identification (*Autos n°* 000…), the action (*Ação: Procedimento do…*), the plaintiff (*Autor: Nome do…*), and the defendant (*Réu: Nome da…*).

In general, after this section the parties will be cited as either plaintiff (*Autor* in Portuguese) or defendant (*Réu* in Portuguese). Thus, this is the main place anonymization is required. Having the specific parties names and the process identification, we searched over the text for them and also removed any of their occurrences.

For the anonymization process, we design an automatic algorithm to detect the parties name and the process identification. To do so, we used Regular Expressions which are patterns we ought to find in the text.

In Table A1, we present the patterns to find the parties and the process identification and the corresponding value we chose as a substitute. However, we used different approaches for parties and the process identification. For the former, *i.e.,* plaintiff and defendant, we tried to find the position (line in the document) where the names were written and then replace all the text after that position. This was required as names do not have a specific pattern but the text that proceeds them do. Having the specific names, we also replaced them over the text. The process identification, on the other hand, had two possible patterns. Thus, we used regular expressions to find and replace the text.

**Table A1:** Patterns for findings the entities in the text and their corresponding.

Entity	Patterns	Type	Substitute
Plaintiff	"autor:"	Position	AUTOR
	"requerente:"		
	"requerente(s):"		
	"autora:"		
	"requerentes:"		
	"autores:"		
	"autoras:"		
Defendant	"réu:"	Position	REU
	"ré:"		
	"requerida:"		
	"requerido:"		
	"requeridas:"		
	"requeridos:"		
	"requerido(a)(s):"		
	"rés:"		
	"réus:"		
Process identification	[0-9]+.[0-9]+.[0-9]+-[0-9]+[0-9]+-[0-9]+.[0-9]+.[0-9]+.[0-9]+.[0-9]+	Replace	NUMERO_PROC

The selection of expressions for finding the entities was done in a iterative manner with the help of a legal expert. Firstly the legal expert analyzed the documents to define the starting expressions to detect the entities. Then we run the algorithm using such patterns. However, due to small differences in writing among the documents, not all documents fitted the patterns. Therefore, the legal expert tried to find the missed expressions and added it to the list of possible expressions for each document. For this reason, the process was repeated until all the documents were correctly anonymized.

As a way to ensure that all the documents were correctly anonymized, we created a new dataset of the documents which successfully passed the anonymization process. And when the new dataset reached the total number of documents of the original dataset, we considered it as completed anonymized.

## Supplemental Information

10.7717/peerj-cs.1225/supp-1 Supplemental Information 1 Source code.

10.7717/peerj-cs.1225/supp-2 Supplemental Information 2 JEC Judgments.

## Additional Information and Declarations

### Data Availability

The following information was supplied regarding data availability: The anonymized data and code are available in the Supplemental Files.
