# Supplementary figures and images for: Regression applied to legal judgments to predict compensation for immaterial damage
Source: PeerJ Comput Sci. 2023 Mar 23;9:e1225. doi: 10.7717/peerj-cs.1225 (PMC10280496; doi:10.7717/peerj-cs.1225)

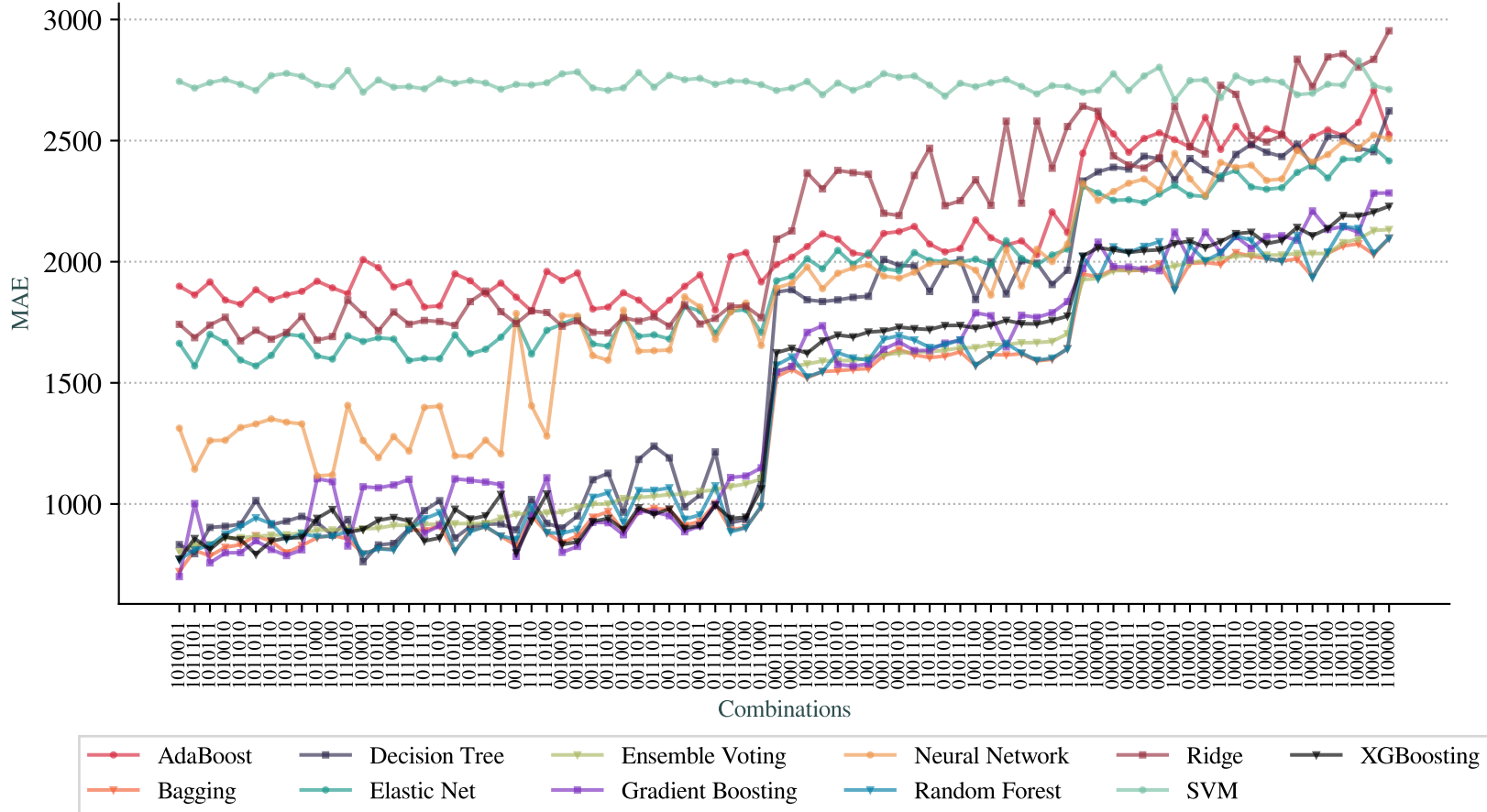

Supplement: Supplemental Information 1 [file peerj-cs-09-1225-s001.zip › data/paper/final_analysis/combinations_mae.pdf]

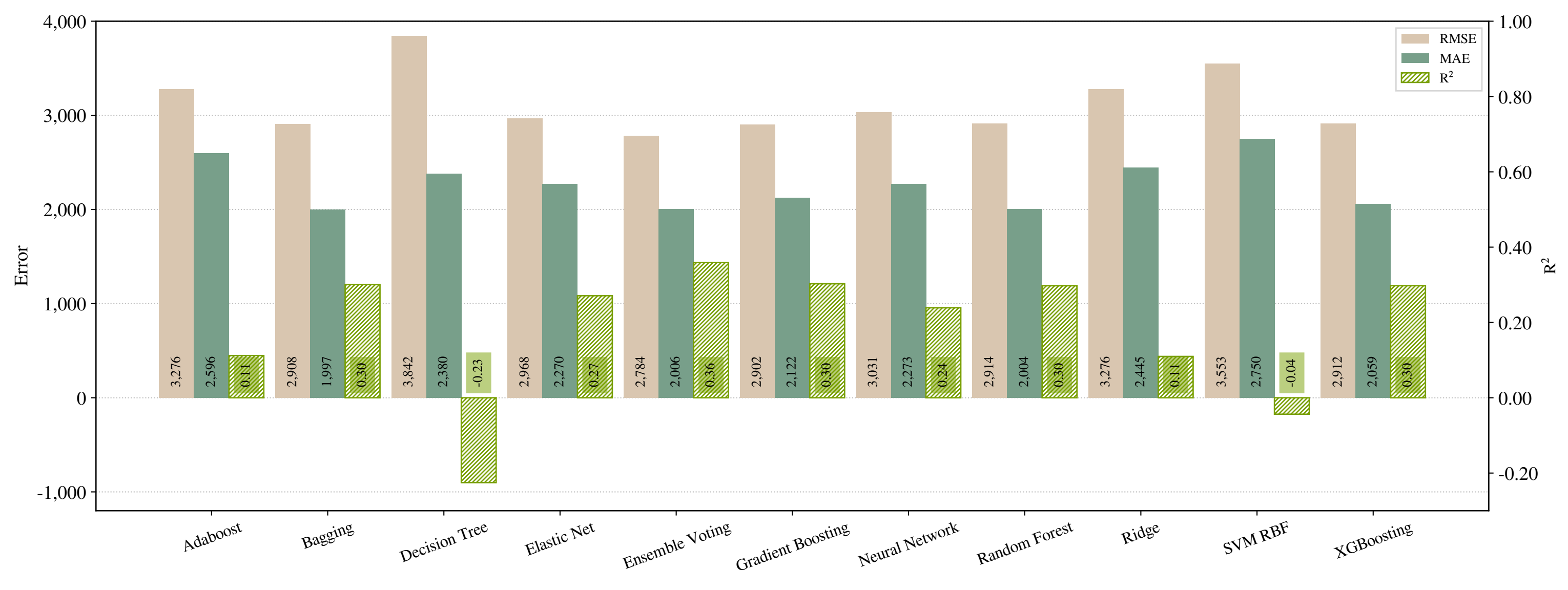

Supplement: Supplemental Information 1 [file peerj-cs-09-1225-s001.zip › data/paper/final_analysis/results_regression_wo_fs_tf_w_or1_wo_ng_wo_at_wo_cv_wo_oa_wo_or2_r2_rmse_test.pdf]

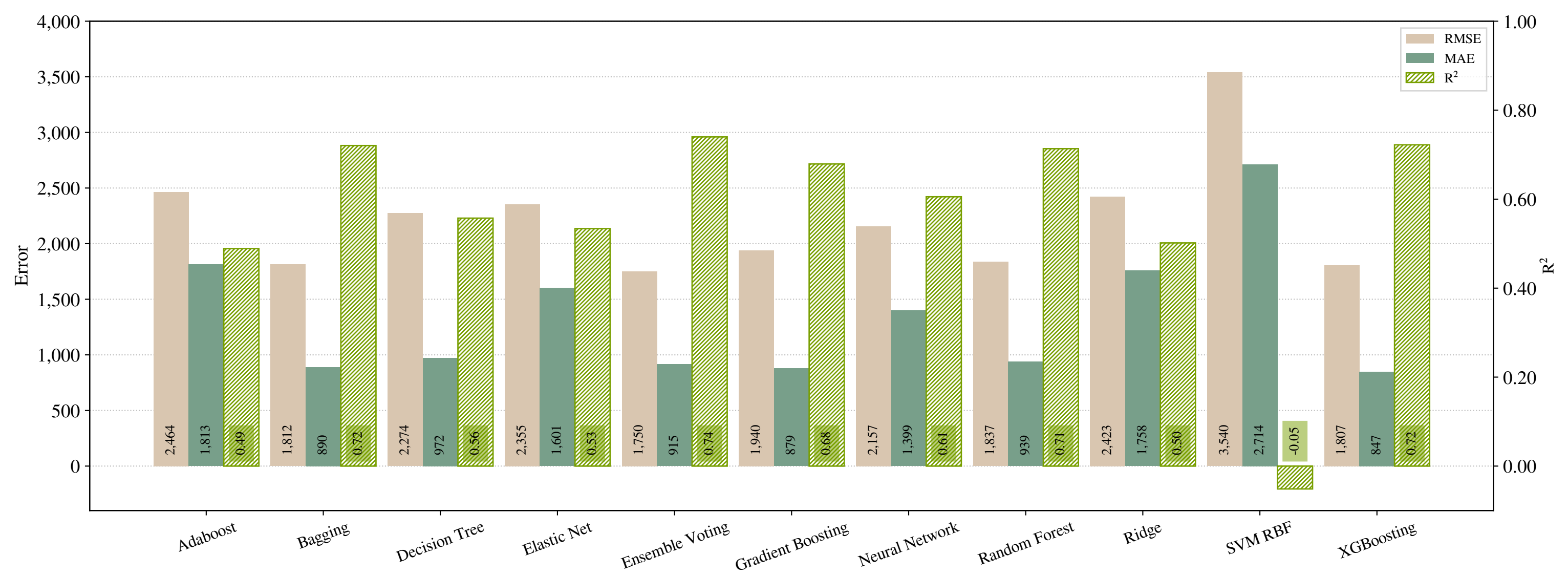

Supplement: Supplemental Information 1 [file peerj-cs-09-1225-s001.zip › data/paper/final_analysis/results_regression_w_fs_before_500_tf_w_or1_w_ng_w_at_w_cv_w_oa_w_or2_r2_rmse_test.pdf]

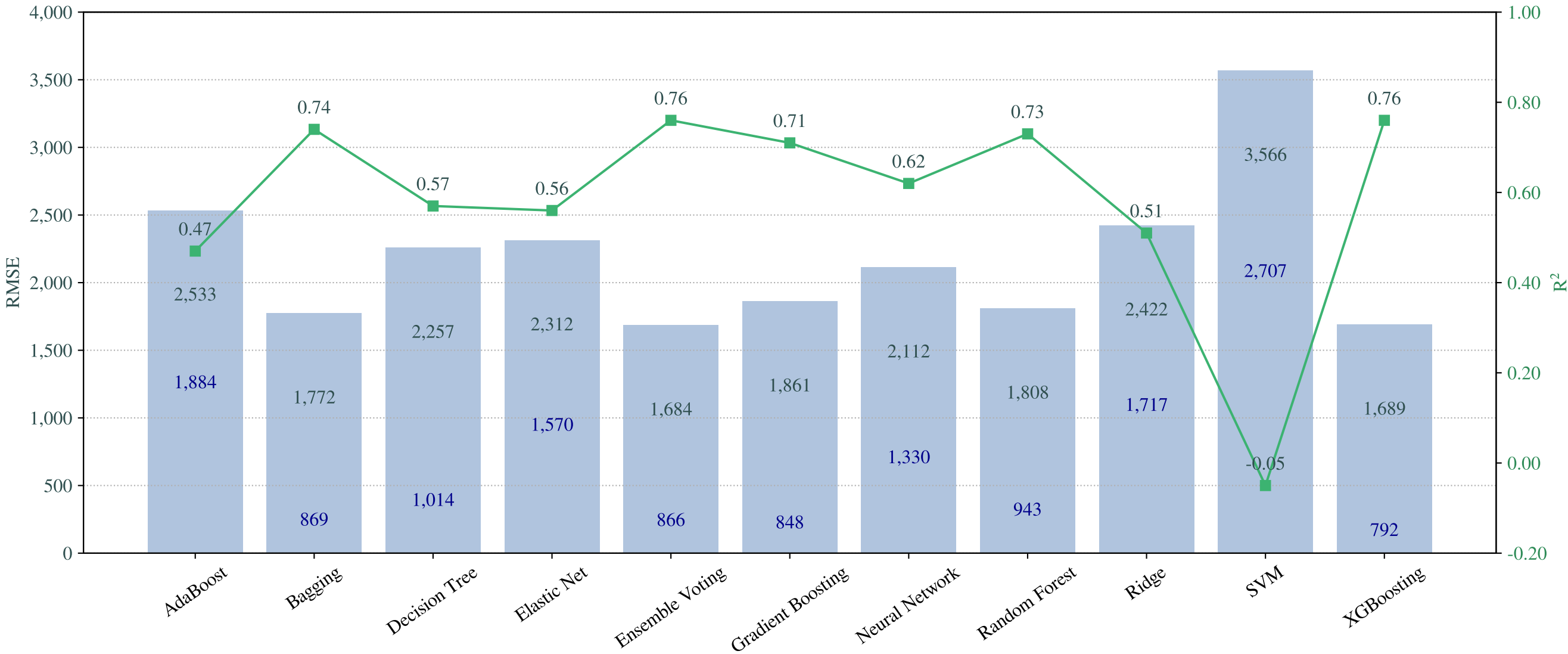

Supplement: Supplemental Information 1 [file peerj-cs-09-1225-s001.zip › data/paper/final_analysis/best_pipeline_r2_rmse.pdf]

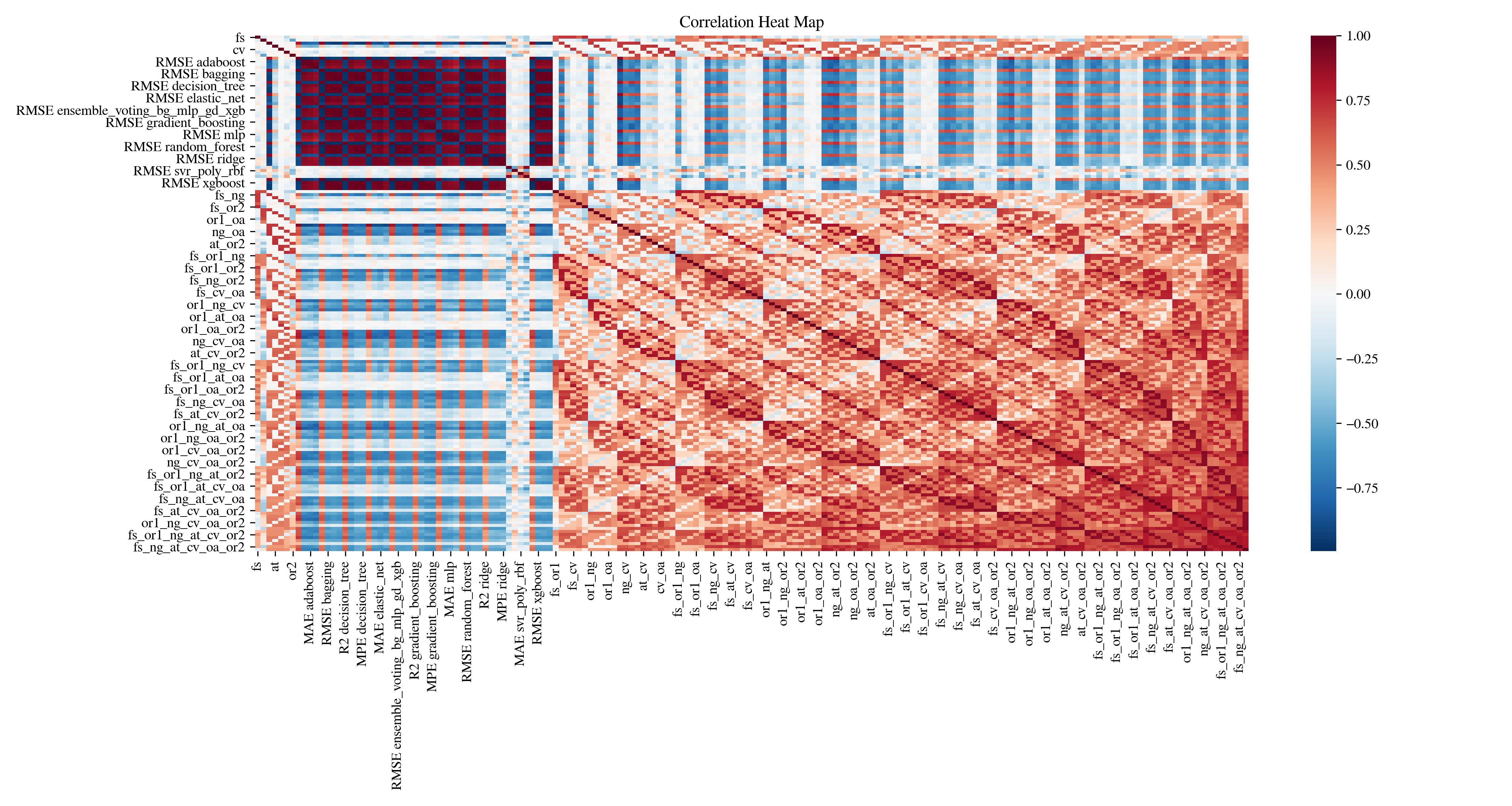

Supplement: Supplemental Information 1 [file peerj-cs-09-1225-s001.zip › data/paper/final_analysis/correlation_heatmap.png]

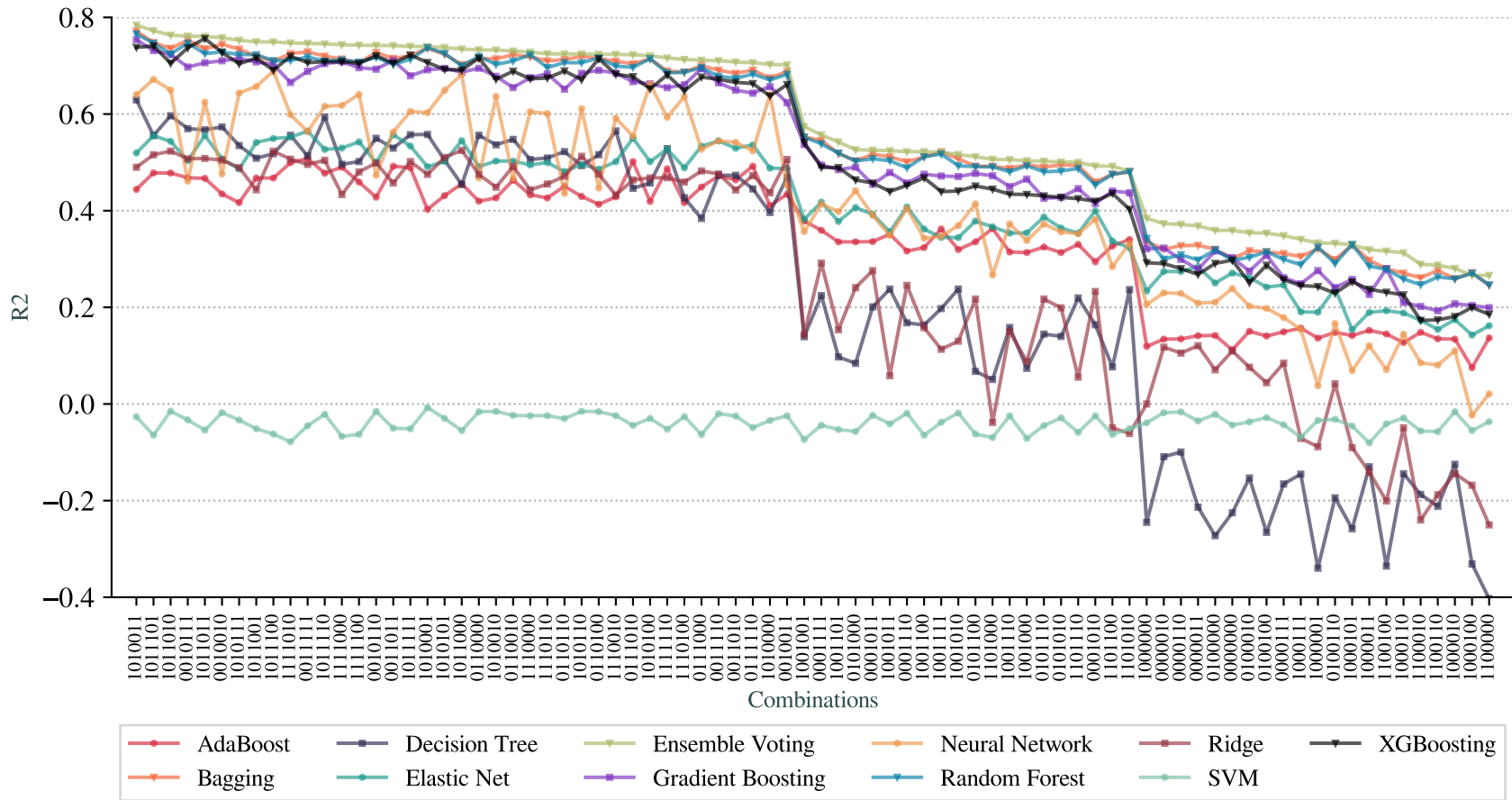

Supplement: Supplemental Information 1 [file peerj-cs-09-1225-s001.zip › data/paper/final_analysis/combinations_r2.pdf]

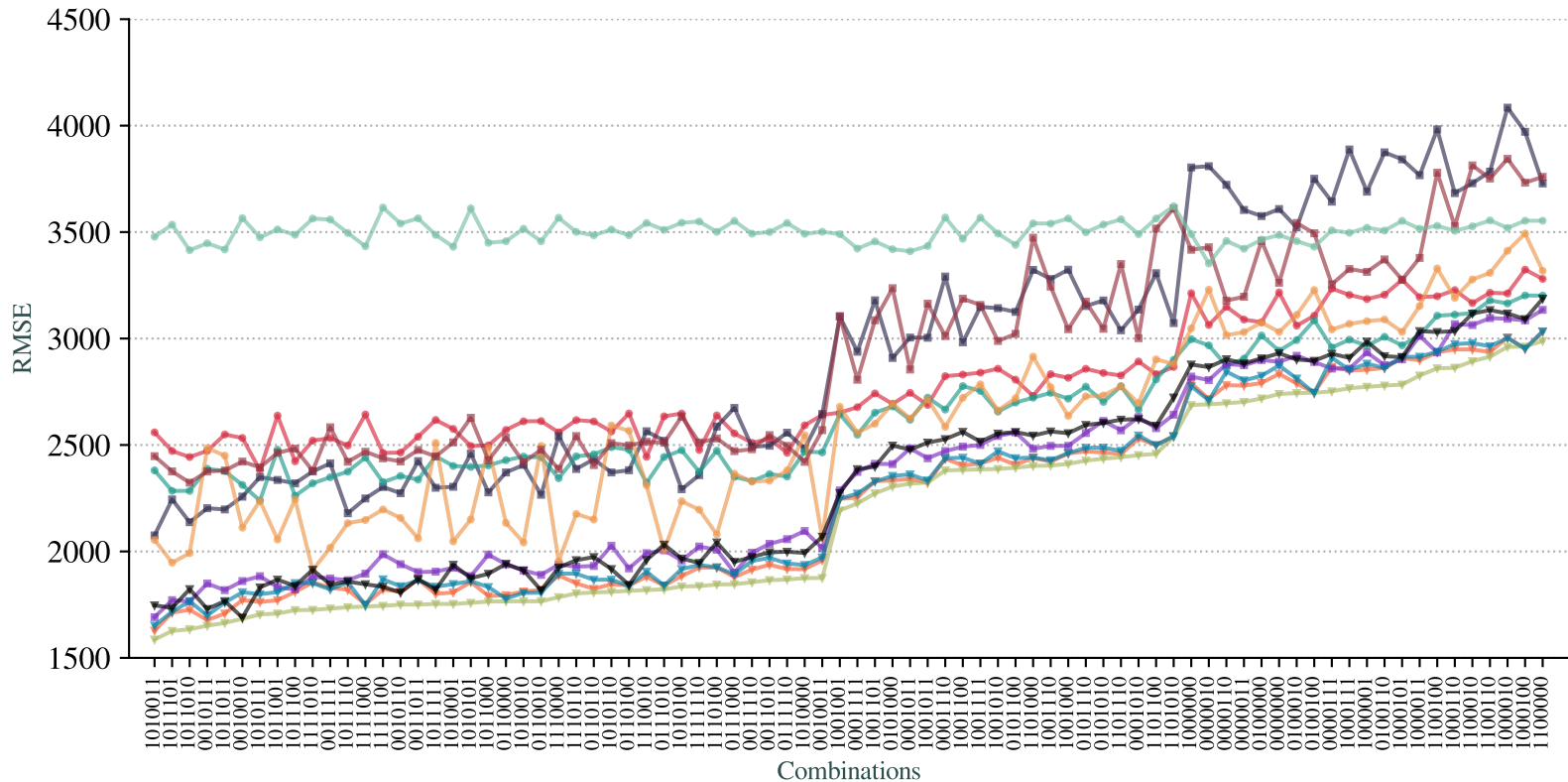

Supplement: Supplemental Information 1 [file peerj-cs-09-1225-s001.zip › data/paper/final_analysis/combinations_rmse.pdf]

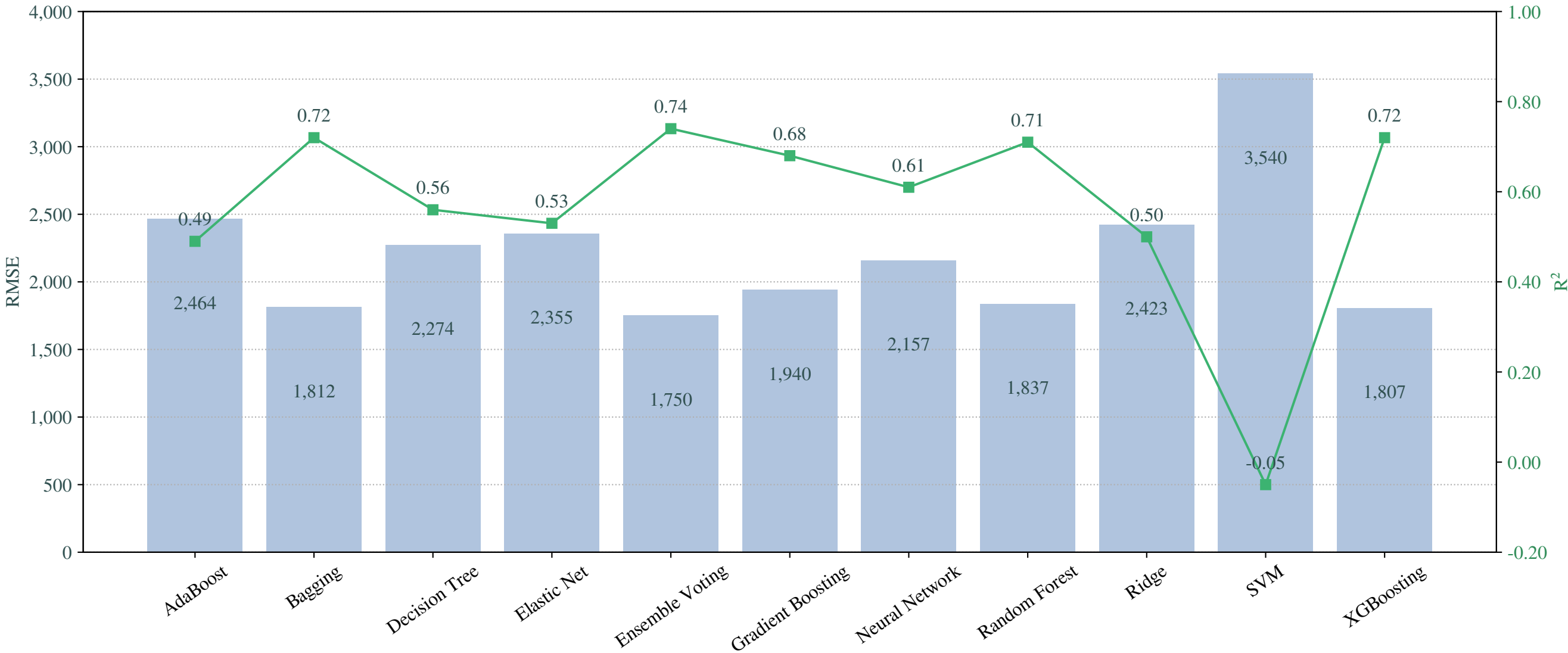

Supplement: Supplemental Information 1 [file peerj-cs-09-1225-s001.zip › data/paper/final_analysis/full_pipeline_r2_rmse.pdf]
